# Advancements in Electron Diffraction: A broad overview of the current state of ELDICO *ED-1* electron diffractometer and some case studies.

**DOI:** 10.1063/4.0001092

**Published:** 2025-10-27

**Authors:** Danny Stam, Gustavo Santiso-Quinones, Christian Jandl, Johannes Merkelbach, Laura Samperisi

**Affiliations:** 1 ELDICO Scientific AG, Allschwil, Basel-Land, Switzerland; 2 ELDICO Scientific AG, Allschwil, basel-land, Switzerland

## Abstract

3D ED / microED has been rapidly evolving as a complementary technique for SC-XRD experiments.[1] Continuous rotation ED experiments work in the same manner as SC-XRD experiments but overcome the critical bottle-neck problem: crystal size. However, the new bottleneck now lies in instrumentation. Dedicated electron diffractometers[2] make the technique more accessible, improving the ease of use and allowing broader application. In fact, in the last 3 years, there has been an increase in the number of publications arising from such devices.[3]

We would like to showcase, not only the technology, but emphasize the advantages and capabilities of having a dedicated device for electron diffraction experiments. Moreover, we would compare the technology to existing technologies like SC-XRD or XRPD, highlight new fields of applications like “crystal mapping” for phase or impurity identification and present some studies where ED has been a game changer for industrial applications.[3b, 4]
